# Facilitative and Inhibitory Effect of Litter on Seedling Emergence and Early Growth of Six Herbaceous Species in an Early Successional Old Field Ecosystem

**DOI:** 10.1155/2014/101860

**Published:** 2014-07-01

**Authors:** Qiang Li, Pujia Yu, Xiaoying Chen, Guangdi Li, Daowei Zhou, Wei Zheng

**Affiliations:** ^1^Northeast Institute of Geography and Agroecology, Chinese Academy of Sciences, 4888 Shengbei Street, Changchun 130102, China; ^2^University of Chinese Academy of Sciences, Beijing 100049, China; ^3^Graham Centre for Agricultural Innovation, and New South Wales Department of Primary Industries, Charles Sturt University, Wagga Wagga, NSW 2650, Australia

## Abstract

In the current study, a field experiment was conducted to examine effects of litter on seedling emergence and early growth of four dominant weed species from the early successional stages of old field ecosystem and two perennial grassland species in late successional stages. Our results showed that increased litter cover decreased soil temperature and temperature variability over time and improved soil moisture status. Surface soil electrical conductivity increased as litter increased. The increased litter delayed seedling emergence time and rate. The emergence percentage of seedlings and establishment success rate firstly increased then decreased as litter cover increased. When litter biomass was below 600 g m^−2^, litter increased seedlings emergence and establishment success in all species. With litter increasing, the basal diameter of seedling decreased, but seedling height increased. Increasing amounts of litter tended to increase seedling dry weight and stem leaf ratio. Different species responded differently to the increase of litter. *Puccinellia tenuiflora* and *Chloris virgata* will acquire more emergence benefits under high litter amount. It is predicted that *Chloris virgata* will dominate further in this natural succession old field ecosystem with litter accumulation. Artificial *P. tenuiflora* seeds addition may be required to accelerate old field succession toward matured grassland.

## 1. Introduction

The emergence and early seedling growth are two crucial processes for establishment and performance of plants [1, 2]. For species in natural communities, a successful emergence not only implies that seedling finally breaks through the soil surface but also emphasizes the importance of emergence time and rate. Previous studies suggested that small difference in the emergence order of plants could determine their final fates under ubiquitous competition [3, 4]. Generally, during the establishment of seedling in community, earlier emergence can be a good trait for plants, because the quicker emergence can help plants occupy the priority of resource utilization, including light, soil water, and nutrition [5]. Apart from emergence, the early seedling growth also has great importance for the establishment and performance of plants [2]. In shorter time after emergence, the seedlings with relatively faster growth rate can approach greater plant size which helps it to occupy wider niche [6], consequently to acquire more advantage in competition for resource, particularly when the resources are limited [7].

Seedling emergence and early growth can be impacted by various factors, such as the seed characteristics [8, 9], seed position in soil profile [10, 11], environment condition such as climate [12], soil physical and chemical properties [12], and biological interference [11]. Increasing evidence suggests that the environment condition plays important role in regulating the emergence and early growth of seedling, which not only acts directly on seedling emergence and growth process but also modifies the effects of other factors on these two processes [13–16].

The amount of litter induced by land use change is an important environmental factor for global plant system [17, 18], which may control species recruitment and affect the structure and dynamics of plant communities [19, 20]. Effects of litter on seedling emergence and growth have largely been reported [17, 18, 21]. Litter can promote the seed germination and seedling growth by keeping soil temperature and moisture with ground cover and increase soil nutrient through decomposition [21–23]. However, the litter also may be disadvantageous for seedling emergence and growth with regard to reducing the light radiation to the soil surface [24], forming mechanical barrier [19, 25], or possibly releasing toxic secondary metabolites [26, 27]. The net effect of litter on seedling emergence and growth is the balance between facilitative and inhibitory actions. Previous studies showed that this “net effect” was controlled by the ecosystem type, litter amount, seed and seedling characteristics, and experiment method (greenhouse versus field) [11, 17, 18, 28]; therefore, for better understanding of the roles of litter in regulating emergence and early growth of seedling, it is necessary to further run experiments under different ecosystem types and experimental methods.

The abandonment of reclaimed grassland occurs globally due to degradation and declined yield, which causes old fields to form. These old fields will renewably be converted into grassland after going through long time natural succession [29–31]. Once abandoned, the old fields are occupied by natural vegetation, which increases litter cover [29, 32] and, subsequently, induces change in surface soil environment [33], which may have important effects on plant establishment and species recruitment. During early successional stage, these old fields generally are dominated by some pioneer species (normally volunteer weed) which reserve a large number of seeds in soil. The fate of these seeds will increase litter cover and influence the further community assembly greatly [34].

In Songnen plain of northeast China, due to arid and soil alkalization, large area of croplands were abandoned and became old fields. The restoration of these old fields ecosystem has important ecological and economic significance for this region. The soil moisture and alkali are two important factors to regulate old field succession, and we expect that these two factors can be improved by increasing litter amount. We designed this experiment to examine how varying litter covers impact the soil properties, emergence features including emergence time and rate, and early growth of seedlings from four dominant weed species in early successional stage and two perennial species in late successional stage in this old field ecosystem. We expected that our study will provide further understanding for the relationship between litter cover and plant establishment and also support a decision tool making to restore this old field ecosystem toward grassland.

## 2. Materials and Methods

### 2.1. Study Site

This study was conducted at Grassland Farming Research Station (E123°31′, N44°33′; Elevation 145 m) of Northeast Institute of Geography and Agroecology, Chinese Academy of Sciences, which is located at the Songnen Plain of northeast China. This study site has semiarid with continental climate. Mean annual temperature is 4.9°C; annual precipitation is approximately 410 mm, with 70% falling from June to September. The soil type is meadow saline-alkali soil, with high soil basic salt content. The typical vegetation in this study site is* Leymus chinensis* meadow. A large area of* L. chinensis* meadow was converted into cropland due to the demand of grain during the last few decades. However, because the decline of soil fertility and crop yield after continuous tillage, some of the reclaimed croplands were abandoned as old field and were expected to restore grassland. In 2011, the current study was conducted on a recent abandoned cropland, two year ago. At the start of the experiment, the site was dominated by a range of annual weed species. The soil bulk density, soil organic carbon, and total nitrogen concentration at the depth of 0–20 cm were 1.47 ± 0.04 g cm^−3^, 10.45 ± 0.58 g kg^−1,^ and 0.98 ± 0.09 g kg^−1^, respectively.

### 2.2. Study Species

We selected four dominant weed species in this old field, including* Abutilon theophrasti* (Malvaceae),* Chenopodium glaucum* (Chenopodiaceae),* Sonchus brachyotus* (Compositae), and* Chloris virgata* (Gramineae), which occupied more than 90% of the aboveground biomass in the community. Among these four species,* A. theophrasti*,* C. glaucum*, and* S. brachyotus* were typical weed species in cropland, while the* C. virgata* was a common species in cropland and natural grassland. Two perennial species from natural grassland,* Puccinellia tenuiflora* (Gramineae) and* Lespedeza davurica* (Leguminosae), which are potentially recruited species in later successional stage of old field (Table 1).

The seeds of each species were collected in autumn 2010 from 10 different populations and at least 10 individuals of each population. Seeds were stored in darkness at room temperature (20°C) until sowing on 26 April 2011. An initial germinating capacity test on additional seed batches was conducted by examining germination rate under optimum light, temperature, and water condition.

### 2.3. Experimental Design

The experiment was a completely randomised block design. There were 4 repeated blocks; 5 litter treatments (0, 200, 400, 600, and 800 g m^−2^) were randomly assigned to each block. Totally, there were 20 plots, and the plot size was 4 m × 4 m with 0.5 m buffers between plots. Within each plot, two microplots (1 m × 1 m) were placed at the centre of the plot, side by side, with 0.5 m space between two plots.

In mid-April 2011, all aboveground plant materials were removed. The soil at the depth of 0–20 cm in all microplots was collected and then steam-sterilised prior to the experiments to kill any plant seeds potentially present in the substrate. The steam-sterilised soil was filled back into original microplots.

On 26 April 2011, one of the microplots, randomly selected, was sowed with 50 seeds of each species, and the other microplot did not receive any seed as control plot. Any germination from control plot was from external seeds. Prior to sowing,* L. davurica* seeds were soaked in 98% H_2_SO_4_ for 0.5 hour to break hard seed coat. The seeds were spread on the soil surface and covered by a thin layer of soil. Immediately after sowing, the whole plot was covered by litters with designated amount according to treatments. The litter was* L. chinensis* hay harvested in adjacent meadow in autumn 2010. The designed litter addition level represented the natural litter production from low to high productivity in the old field ecosystem.

### 2.4. Measurements

#### 2.4.1. Soil Properties

When the soil was filled, a soil water probe and a soil temperature probe, connected on a FDS-100 Automatic Temperature and Moisture Recorder (Handan Electronic Technology Company, Handan, China), were installed at the 5 cm depth in each microplot. Soil temperature and moisture at 5 cm were recorded automatically every 1 hour during the experimental period from 27 April to 7 June 2011. For reflecting soil salinity, soil electrical conductivity (EC) was measured every 6 or 7 days at 0–10 cm on each of the sown microplots using a Field Operated Meter (Easy Test, Poland).

#### 2.4.2. Seedling Emergence

The emerged seedling was checked and marked every day in all microplots from sowing till 7 June 2011. In fact, no new germination was counted after 26 May. At each count day, all new emergent seedlings from 6 study species were marked using plastic label with species name and emergence date. The seedling emergence was defined as seedling successfully penetrated through the litter cover.

#### 2.4.3. Early Seedling Growth

On 8 June 2011, the marked seedlings for each species were counted, and seedling mortality was recorded in each sown plot. Five seedlings according to emergence sequence for each species were randomly selected from each microplot to measure height and basal diameter. Each seedling was divided into stem and leaf (with petiole) to determine dry weight after being oven-dried at 70°C for 48 h.

### 2.5. Data Analysis

Seedling emergence time was the number of days from seed sowing to seedling emergence [35]. Seedling emergence rates were represented using the Emergence Rate Index (ERI) as described by Erbach [36]. The ERI is calculated using the following:

(1) $$\begin{array}{c} \text{ERI}=\overset{\text{LD}}{\underset{\text{FD}}{\sum }}\frac{P_{n}-P_{\left(n-1\right)}}{N}, \end{array}$$

where *N* is the number of days since planting, *P*_*n*_ is the percentage of plants emerged on day *n*, *P*_(*n*−1)_ is the percentage of plants emerged on day (*n* − 1), LD is the last day when emergence was complete, and FD is the first day counting began. In this study, FD was set at 1.

The emergence percentage was calculated as the ratio of emergence seedling number to germinable seed number. The survival rate of seedling was calculated as ratio of remaining marked seedlings to totally marked seedlings. The establishment success rate for each species was calculated by emergence percentage multiplying the survival rate of seedling [35].

Repeated-measures ANOVA tests were used to examine the effects of litter cover on soil electrical conductivity with time as a fixed factor. A simple lineal regression was used to examine the correlation relationship between soil EC and soil moisture. Two-way ANOVA analysis was applied to determine the main and interactive effects of litter cover and species on seedling emergence, seedling establishment, and seedling growth. The mean comparison was conducted amongst treatments using Duncan's *t*-test after all data were assured to be normal. Significant differences for all statistical tests were evaluated at *P* = 0.05. All data analyses were conducted with the SPSS16.0 software (Chicago, IL, USA).

## 3. Results

### 3.1. Soil Properties

**Figure 1:**
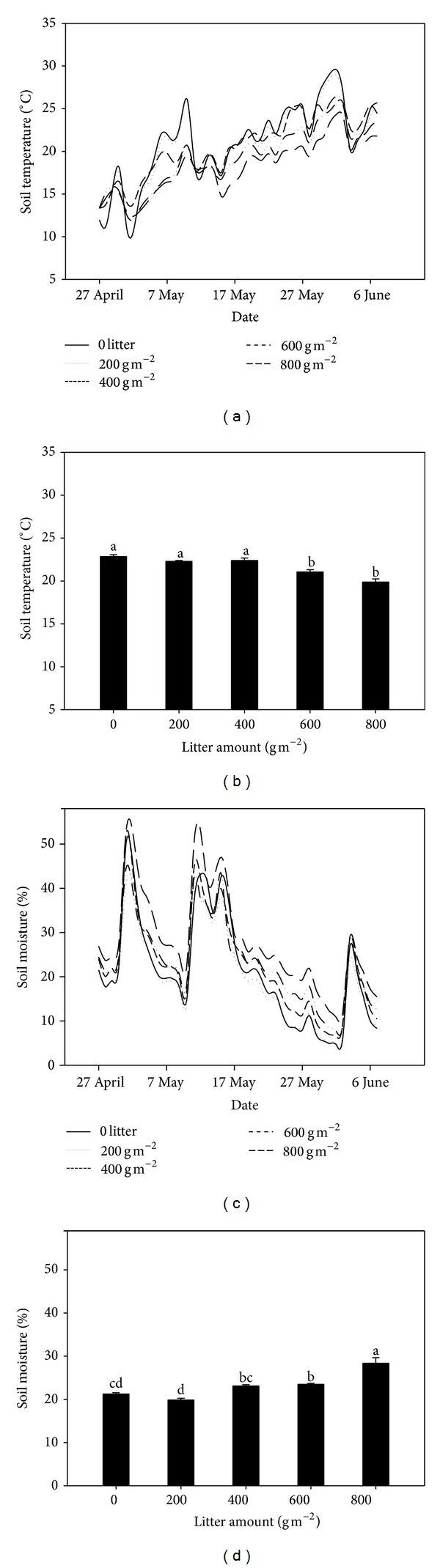
Soil temperature ((a) and (b)) and moisture ((c) and (d)) under different litter cover treatments. The lines represent the temporal dynamic of soil temperature and moisture; the bars with mean + SE represent mean soil temperature and moisture from 27 April 2011 to 7 June 2011; different letters indicate significant difference between treatments at *P* < 0.05.

**Figure 2:**
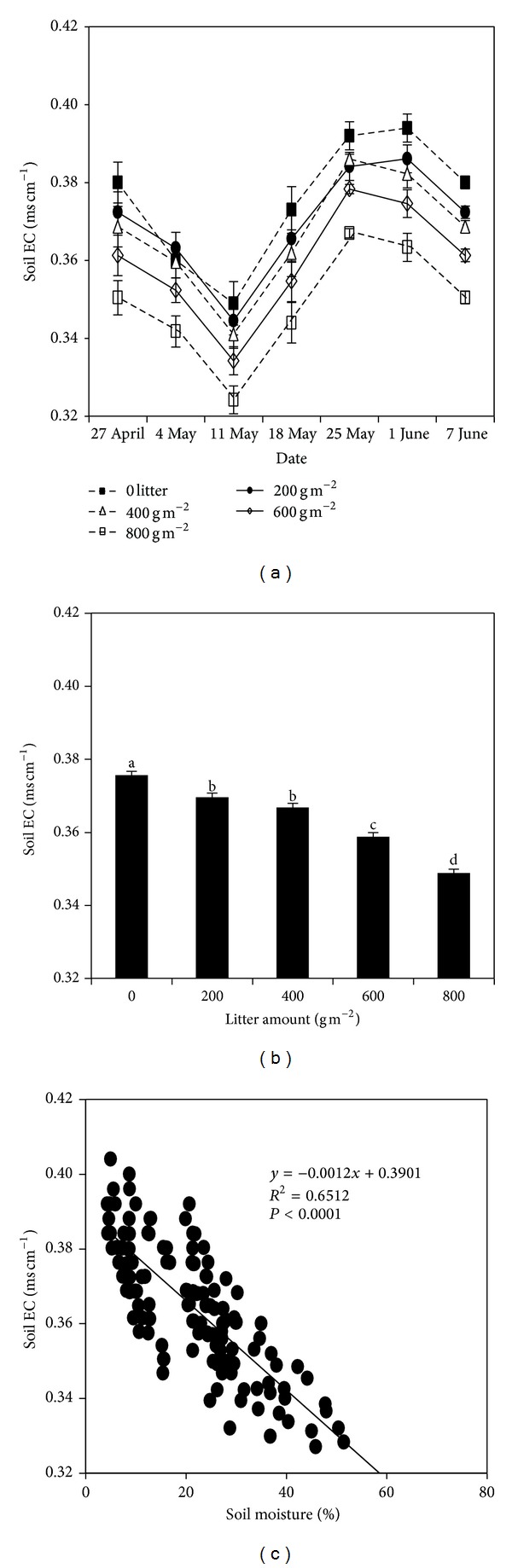
The temporal dynamic (a) and mean values (b) of soil electrical conductivity (EC), and the relationship between soil EC and soil moisture (c); the bars with mean ± SE represent mean soil temperature and moisture from 27 April 2011 to 7 June 2011; different letters indicate significant difference between treatments at *P* < 0.05.

Mean daily soil temperature and moisture (belowground 5 cm) varied greatly over the growing seasons (Figures 1(a) and 1(c)). Mean daily soil temperature increased over time for all treatments but with more fluctuating pattern under lower than high litter cover treatments. The soil temperature gradually decreased as litter cover increased. Mean soil temperature under 600 and 800 g m^−2^ litter cover was significantly lower than 0, 200, and 400 g m^−2^ litter cover treatments (Figures 1(a) and 1(b)). Soil moisture generally increased with increased litter cover. The 800 g m^−2^ litter cover treatment had the highest soil moisture (Figures 1(c) and 1(d)). Soil EC showed a strong temporal pattern (*P* < 0.001), which was significantly influenced by litter cover (*P* < 0.001; Figure 2(a)). Soil EC decreased with increase of litter cover over the experimental period (Figure 2(b)). Regression analysis showed that there was a significantly negative correlation relationship between soil EC and soil moisture (Figure 2(c)).

### 3.2. Seedling Emergence Time, Rate, and Percentage

**Figure 3:**
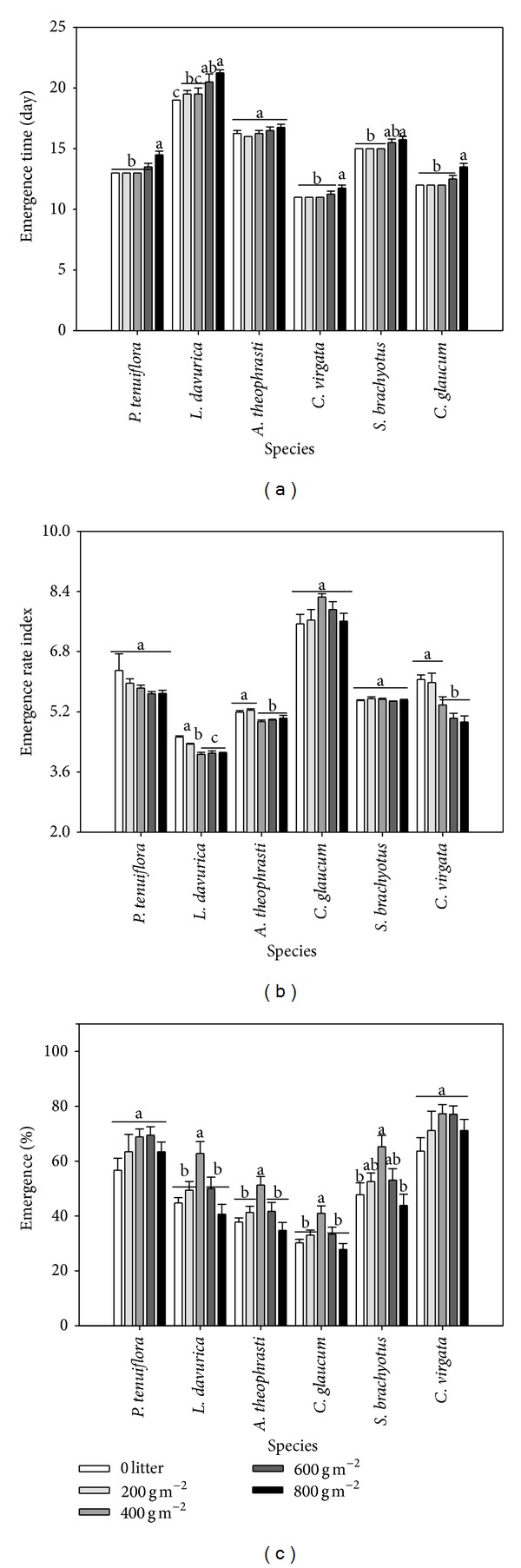
The emergence time (a), emergence rate (b), and emergence percentage (c) of seedling under different species and litter covers. The values were represented as mean + SE; different letters indicate significant difference between treatments at *P* < 0.05.

The seedling emergence time, emergence rate, and emergence percentage of seedlings varied among different litter cover and species. There was significant interaction between litter cover and species on emergence rate of seedlings (Table 2; Figure 3).* C. glaucum* germinated at the earliest time and fastest rate, followed by* P. tenuiflora* and* C. virgata*, and* L. davurica* had the latest emergence time and slowest emergence rate (Figures 3(a) and 3(b)). The increase of litter cover tended to delay seedling emergence time and emergence rate, although slightly higher emergence rate of* C. glaucum* was found with 400 and 600 g m^−2^ than 0 and 200 g m^−2^ litter cover (Figures 3(a) and 3(b)). The emergence time of* L. davurica* seedlings was delayed by two days from 0 to 800 g m^−2^ litter cover (Figure 3(a)). The emergence rate index of* C. virgata* seedlings was decreased by 28% from 0 to 800 g m^−2^ litter cover (Figure 3(b)). Amongst species,* C. virgata* had the highest emergence percentage, followed by* P. tenuiflora*, and* C. glaucum* had the lowest emergence percentage under all litter covers (Figure 3(c)). The emergence percentage of seedlings firstly increased then decreased as litter cover increased, and 400 g m^−2^ litter cover generally had the most positive effect on emergence percentage of seedlings (Figure 3(c)). When litter cover was below 600 g m^−2^, the presence of litter increased seedlings emergence percentage in all species, in particular* P. tenuiflora* and* C. virgata*, whose emergence percentage still was higher under 800 g m^−2^ litter cover than no litter (Figure 3(c)).

### 3.3. Seedling Survival Rate and Establishment Success Rate

**Figure 4:**
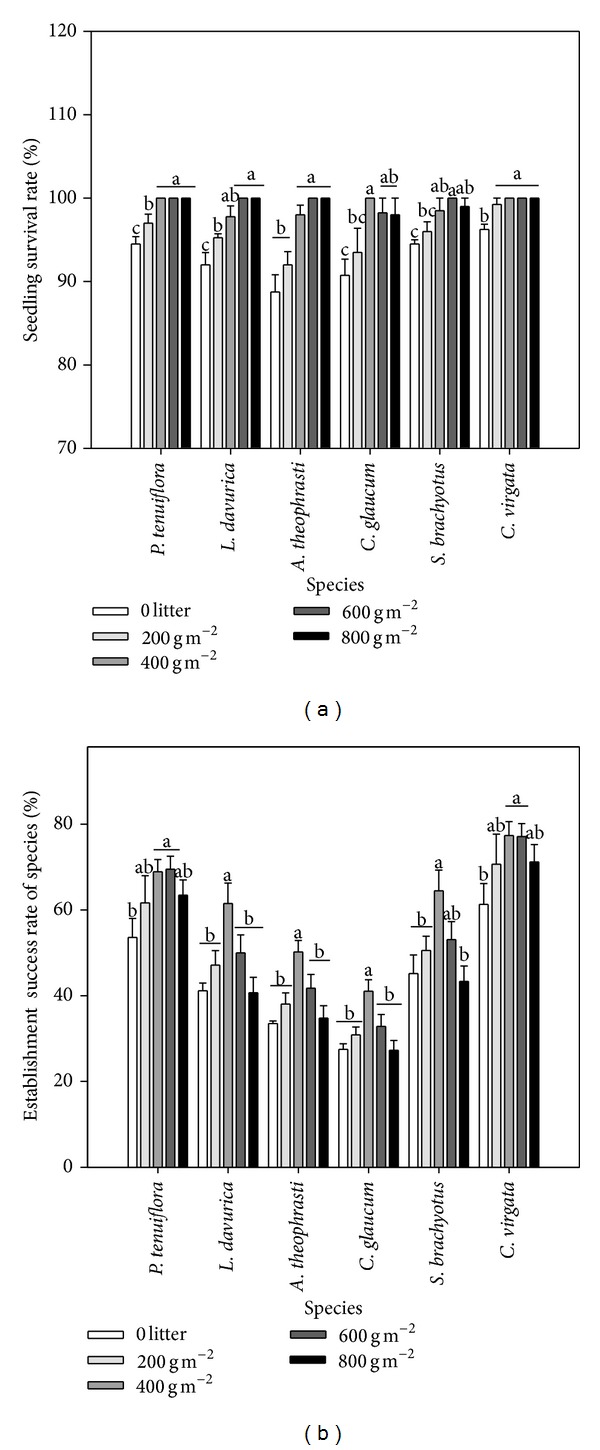
Seedling survival rate (a) and emergence success rate (b) under different species and litter covers. The values were represented as mean + SE; different letters indicate significant difference between treatments at *P* < 0.05.

Two-way ANOVA indicated that litter cover and species had significant impact on the seedling survival rate and establishment success rate (Table 2). The litter cover enhanced the seedling survival rate (Figure 4(a)).* P. tenuiflora* and* C. virgata* had relative higher seedling survival rate compared with other species under all litter covers.* A. theophrasti* had the lowest seedlings survival rate under 0 litter cover compared with other species. As litter cover increases, seedling survival rate increased greatly (Figure 4(a)). The establishment success rate showed similar trend to the emergence percentage of seedling (Figure 4(b)).

### 3.4. Seedling Growth Characteristics

**Figure 5:**
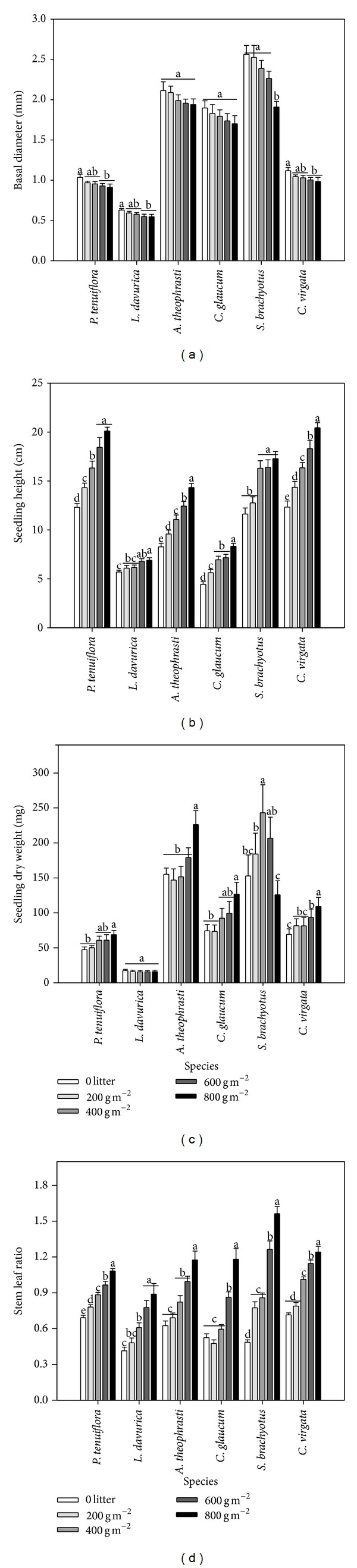
Seedling basal diameter (a), height (b), dry weight (c), and stem leaf ratio (d) under different species and litter cover. The values were represented as mean + SE; different letters indicate significant difference between treatments at *P* < 0.05.

There were significant interactions in growth characteristics of seedling between litter cover and species (Table 2). Increase of litter cover resulted in decrease in basal diameter of seedling but increase in seedling height (Figures 5(a) and 5(b)). In all species,* A. theophrasti* and* S. brachyotus* had the highest seedling dry weight, followed by* C. glaucum* and* C. virgata*, and* L. davurica* had the lowest among all litter covers. High litter cover tended to increase seedling dry weight in most species except for* S. brachyotus* which showed a significant decrease in seedling dry weight from 400 to 800 g m^−2^ litter cover and* L. davurica* which showed no change with litter cover (Figure 5(c)). The stem leaf ratio for all species generally increased as litter cover increased. The stem leaf ratio under 800 g m^−2^ was significantly higher than other litter cover treatments for all species (Figure 5(d)).

## 4. Discussion

### 4.1. The Influence of Litter on Soil Environment

Litter intercepts incident light and rain and changes the surface structure, hence, affecting the transfer of heat and water between the soil and the atmosphere [21], which can greatly influence soil temperature and moisture. Our results showed that increasing litter cover reduced soil temperature and increased temperature variability over time, which is consistent with previous researches [21, 24]. As Facelli and Pickett explained [37], litter intercepted incoming solar radiation and outgoing longwave radiation, which forms an insulating layer for soil to avoid direct heating from solar and heat absorption from atmosphere. Litter cover can improve soil moisture status as evidenced by increased soil moisture with increased litter cover in the current study. Murphy et al. reported that increase litter cover improves water infiltration and reduce water evaporation, which can be helpful for maintaining soil moisture [23].

Soil drought and alkalization are two primary factors to limit plant establishment and growth. At the study site, surface soil drought and alkalization generally simultaneously occur due to the rising of soluble salt from deep soil layer with water transpiration. Results showed that surface soil EC was negatively correlated to increased soil moisture and the increase of litter cover reduced surface soil EC, indicating that high litter cover can increase water infiltration and decrease water evaporation, hence, reducing soil salinity simply by keeping salt in deeper soil layer. The changes of soil temperature, moisture, and salinity due to litter cover may facilitate plant emergence and establishment [38]. On the other hand, however, litter cover can reduce the quantity and quality of light (e.g., the red : far-red ratio) experienced by seeds and seedlings [37] or forms physical obstruction to seedlings growth [25], which may negatively act on emergence and establishment of plants [17, 21].

### 4.2. The Effects of Litter on Emergence and Early Growth of Seedling

The balance between facilitative and inhibitory effects of litter on seedlings emergence and growth depends on the amount of litter cover [39, 40]. Moderate litter covers may support seedlings emergence and growth by improving soil microclimate conditions [24, 41, 42]. However, facilitative effects are reduced when amount of litter covers are too high [17, 18], because high litter cover reduces light quantity and quality to cause deep shade or darkness [24, 41] and may create an impenetrable physical barrier for seedlings [37]. Loydi et al. found that litter cover had positive effects on emergence in grassland ecosystems when litter was below 500 g m^−2^ and seedling survival and biomass increased with <250 g m^−2^ litter cover [18]. Our results showed that emergence percentage and establishment success rate of all species increased when litter cover was below 600 g m^−2^, and no significant differences were found between 800 g m^−2^ and 0 litter treatments, in particular for two grass species (Figures 3(c) and 4(b)). Nevertheless, the emergence percentage and establishment success rate of* P. tenuiflora* and* C. virgata* still were higher under 800 g m^−2^ litter cover than no litter. Moreover, in our study, even under high litter cover, the seedlings survival rate of all species was higher than 0 litter treatment, and increasing litter cover tended to increase seedling biomass of all species except* S. brachyotus* (Figure 5(c)). In addition, more litter can greatly increase soil moisture and reduce soil salinity. These results indicate that the facilitative effects of increasing litter cover are more important than its inhibitory effects at our study site.

The emergence time and rate may determine the plant sequence in resource utilization, which can influence the plant fate in community, in particular when resources are limited [5]. Our study indicated that high litter cover delayed the emergence time of all species and reduced the emergence rate of most species except for* C. glaucum* (Figure 3(b)), as seedlings needed more time to penetrate a thick litter layer.

The change of seedling morphology reflects the plant adaptability to environment conditions [43]. Our results showed that the basal diameter of seedlings decreased, but seedlings height and stem leaf ratio increased as litter cover increased. It was because increasing litter cover enhances the obstruction for seedling emergence [25] and also decreased the near-surface light availability [37], which caused seedlings to invest more energy to stem for upward growth to penetrate litter and intercept light [44], consequently inducing more biomass allocation to stem. The reduced seedling basal diameter has advantage for seedlings to pass through small gap under dense litter, which may be an effective adaptive strategy for plants subjected to thick litter cover.

### 4.3. The Responses of Emergence and Early Growth for Different Species to Litter Cover Change

Different species respond to litter cover differently in terms of seedling emergence rate and early growth. Seed size was considered as a good predictor for the effect of litter [28, 45, 46]. Loydi et al. showed that litter had stronger negative effects on emergence, survival, and biomass of seedlings from smaller seed (<1 mg) but slight positive effects on species with bigger seed (>1 mg) [18]. Relevant mechanisms were proposed to explain these differences, including light requirement of small seed species during germination process [47], the reserve effect [48], seedling size effect [49, 50], and the metabolic effect [18] related to seed size. In our study, the effect of litter on emergence rate and seedling growth varied between species, but no orderly species responses were found to be related to seed sizes. Regardless the seed size, we did not observe any trend of seedling emergence rate and growth related to life form or source habitat. Compared with previous studies, our results either were because there may be more complicate interrelations between environments and plants to control the effect of litter in this old field ecosystem or the study species is too few to reflect a general rule.

Although no general relationships presented between seedling emergence, growth, and species properties, our results showed that* P. tenuiflora* and* C. virgata* had relative higher emergence percentage, survival rate of seedling, and establishment success rate compared with other species (Figures 3(c) and 4). The emergence and seedling biomass of these two species showed more positive response to high litter cover than most other species, which may attribute to their subuliform morphology in earlier seedling stage. As typical weed species, although* A. theophrasti*,* C. glaucum*, and* S. brachyotus* had high seedling biomass (Figure 5(c)), their emergence percentage and establishment success rate were relatively lower than all other species (Figures 3(c) and 4(b)), which were strongly influenced by high litter cover. The emergence rate and establishment success rate of* L. davurica* were higher than* A. theophrasti* and* C. glaucum*, however, its seedlings performance was the worst among all species (Figure 5). This result indicated that* C. virgata* will dominate further in this naturally successional old field ecosystem as litter accumulation increases. First,* C. virgata* will keep higher emergence and establishment capacity under high litter cover than other three weed species. Secondly,* C. virgata* can produce smaller but more seeds. Lastly, the seeds of* C. virgata* mature and fall in early July before new litter is formed (Table 1). As a result, the* C. virgata* seeds can easily pass through litter layer and form soil seed bank [11, 28]. In contrast, the seeds from other three weed species are bigger than* C. virgata*, and these seeds commonly mature and fall after mid-August when more litter has fallen, which may greatly reduce the opportunity for seeds to contact with soil, consequently having less opportunities to germinate and establish compared with* C. virgata*.

## 5. Conclusions

The increase of litter cover can increase soil moisture and decrease surface soil salinity. Increased litter cover can delay seedlings emergence time and rate. Litter cover below 600 g m^−2^ can promote the seedling emergence and establishment of all studied species due to improved soil moisture conditions. Different species respond differently with increased litter cover.* P. tenuiflora* and* C. virgata* will acquire more emergence benefits under high litter cover. Therefore, it is expected that this old field ecosystem possibly becomes* C. virgata* dominated annual grass grassland under natural succession. With respect to similar emergence performance and seed features with* C. virgata*,* P. tenuiflora* may be potential to invade and establish into this old field ecosystem and accelerate the old field succession toward mature grassland, but the seed dispersal limitation may impede the invasion of* P. tenuiflora*; therefore, an artificial seed addition may be necessary.

## Figures and Tables

**Table 1:** Habitat, life form, family, mass per seed (mg), and germinating capacity of each species included in the study.

Habitat	Life form	Species	The start time of seed ripening and falling	Mass per seed (mg)	Germinating capacity (%)
Grassland	Perennial	*P. tenuiflora *	Early July	0.562 ± 0.052	82
	Perennial	*L. davurica *	Late August	2.053 ± 0.044	86

Old field	Perennial	*S. brachyotus *	Mid-August	1.138 ± 0.017	94
	Annual	*A. theophrasti *	Mid-August	8.799 ± 0.125	92
	Annual	*C. glaucum *	Mid-August	0.475 ± 0.028	92

Grassland, old field	Annual	*C. virgata *	Early July	0.337 ± 0.012	84

**Table 2:** The *F* and *P* values of two-way ANOVA analysis for litter cover, species, and their interactions on seedling emergence, seedling establishment, and seedling growth.

Variables	Litter cover		Species		Litter cover × species	
	*F*	*P*	*F*	*P*	*F*	*P*
Emergence time (day)	26.25	<**0.001**	885.28	<**0.001**	1.34	0.117
Emergence rate index	7.11	<**0.001**	325.33	<**0.001**	3.08	<**0.001**
Emergence percentage (%)	15.48	<**0.001**	77.44	<**0.001**	0.68	0.840
Seedling survival rate (%)	43.04	<**0.001**	6.37	<**0.001**	1.63	0.062
Establishment success rate (%)	19.31	<**0.001**	79.59	<**0.001**	0.62	0.892
Basal diameter of seedling (mm)	9.49	<**0.001**	487.26	<**0.001**	1.67	**0.034**
Seedling height (cm)	103.94	<**0.001**	398.22	<**0.001**	4.41	<**0.001**
Seedling dry weight (mg)	6.23	<**0.001**	220.66	<**0.001**	6.10	<**0.001**
Stem leaf ratio	176.85	<**0.001**	48.12	<**0.001**	5.94	<**0.001**

## References

[1] Seiwa K (2000). Effects of seed size and emergence time on tree seedling establishment: importance of developmental constraints. *Oecologia*.

[2] Prasad PVV, Boote KJ, Thomas JMG, Allen LH, Gorbet DW (2006). Influence of soil temperature on seedling emergence and early growth of peanut cultivars in field conditions. *Journal of Agronomy and Crop Science*.

[3] Verdú M, Traveset A (2005). Early emergence enhances plant fitness: a phylogenetically controlled meta-analysis. *Ecology*.

[4] Abe M, Honda A, Hoshizaki K, Miguchi H (2008). Advantage of early seedling emergence in *Fagus crenata*: importance of cotyledon stage for predator escape and pathogen avoidance. *Ecological Research*.

[5] Seiwa K (1998). Advantages of early germination for growth and survival of seedlings of Acer mono under different overstorey phenologies in deciduous broad-leaved forests. *Journal of Ecology*.

[6] Fenner M (2000). *Seeds: The Ecology of Regeneration in Plant Communities*.

[7] Hofmann M, Isselstein J (2004). Effects of drought and competition by a ryegrass sward on the seedling growth of a range of grassland species. *Journal of Agronomy and Crop Science*.

[8] Dalling JW, Hubbell SP (2002). Seed size, growth rate and gap microsite conditions as determinants of recruitment success for pioneer species. *Journal of Ecology*.

[9] Moles AT, Westoby M (2004). Seedling survival and seed size: a synthesis of the literature. *Journal of Ecology*.

[10] Keshtkar E, Kordbacheh F, Mesgaran MB, Mashhadi HR, Alizadeh HM (2009). Effects of the sowing depth and temperature on the seedling emergence and early growth of wild barley (*Hordeum spontaneum*) and wheat. *Weed Biology and Management*.

[11] Donath TW, Eckstein RL (2012). Litter effects on seedling establishment interact with seed position and earthworm activity. *Plant Biology*.

[12] Walters MB, Reich PB (2000). Seed size, nitrogen supply, and growth rate affect tree seedling survival in deep shade. *Ecology*.

[13] Jakobsson A, Eriksson O (2000). A comparative study of seed number, seed size, seedling size and recruitment in grassland plants. *Oikos*.

[14] Wurst S, Langel R, Scheu S (2005). Do endogeic earthworms change plant competition? A microcosm study. *Plant and Soil*.

[15] Ejrnæs R, Bruun HH, Graae BJ (2006). Community assembly in experimental grasslands: suitable environment or timely arrival?. *Ecology*.

[16] Griffith AB, Loik ME (2010). Effects of climate and snow depth on *Bromus tectorum* population dynamics at high elevation. *Oecologia*.

[17] Xiong S, Nilsson C (1999). The effects of plant litter on vegetation: a meta-analysis. *Journal of Ecology*.

[18] Loydi A, Eckstein RL, Otte A, Donath TW (2013). Effects of litter on seedling establishment in natural and semi-natural grasslands: a meta-analysis. *Journal of Ecology*.

[19] Sayer EJ (2006). Using experimental manipulation to assess the roles of leaf litter in the functioning of forest ecosystems. *Biological Reviews of the Cambridge Philosophical Society*.

[20] Eckstein RL, Pereira E, Milbau A, Graae BJ (2011). Predicted changes in vegetation structure affect the susceptibility to invasion of bryophyte-dominated subarctic heath. *Annals of Botany*.

[21] Facelli JM, Pickett STA (1991). Plant litter: its dynamics and effects on plant community structure. *Botanical Review*.

[22] Brearley FQ, Press MC, Scholes JD (2003). Nutrients obtained from leaf litter can improve the growth of dipterocarp seedlings. *New Phytologist*.

[23] Murphy SR, Lodge GM, Harden S (2004). Surface soil water dynamics in pastures in northern New South Wales. 3. Evapotranspiration. *Australian Journal of Experimental Agriculture*.

[24] Jensen K, Gutekunst K (2003). Effects of litter on establishment of grassland plant species: the role of seed size and successional status. *Basic and Applied Ecology*.

[25] Bosy JL, Reader RJ (1995). Mechanisms underlying the suppression of forb seedling emergence by grass (Poa pratensis) litter. *Functional Ecology*.

[26] Bonanomi G, Sicurezza MG, Caporaso S, Esposito A, Mazzoleni S (2006). Phytotoxicity dynamics of decaying plant materials. *New Phytologist*.

[27] Herranz JM, Ferrandis P, Copete MA, Duro EM, Zalacaín A (2006). Effect of allelopathic compounds produced by *Cistus ladanifer* on germination of 20 Mediterranean taxa. *Plant Ecology*.

[28] Miglécz T, Tóthmérész B, Valkó O, Kelemen A, Török P (2013). Effects of litter on seedling establishment: an indoor experiment with short-lived Brassicaceae species. *Plant Ecology*.

[29] Zhao WZ, Xiao HL, Liu ZM, Li J (2005). Soil degradation and restoration as affected by land use change in the semiarid Bashang area, northern China. *Catena*.

[30] McLauchlan KK, Hobbie SE, Post WM (2006). Conversion from agriculture to grassland builds soil organic matter on decadal timescales. *Ecological Applications*.

[31] Wang C, Butterbach-Bahl K, Han Y (2011). The effects of biomass removal and N additions on microbial N transformations and biomass at different vegetation types in an old-field ecosystem in northern China. *Plant and Soil*.

[32] Kosmas C, Gerontidis S, Marathianou M (2000). The effect of land use change on soils and vegetation over various lithological formations on Lesvos (Greece). *Catena*.

[33] Zhang J, Song C, Yang W (2007). Effects of cultivation on soil microbiological properties in a freshwater marsh soil in Northeast China. *Soil and Tillage Research*.

[34] Török P, Miglécz T, Valkó O (2012). Recovery of native grass biodiversity by sowing on former croplands: is weed suppression a feasible goal for grassland restoration?. *Journal for Nature Conservation*.

[35] Ranal MA, De Santana DG (2006). How and why to measure the germination process?. *Revista Brasileira de Botanica*.

[36] Erbach DC (1982). Tillage for continuous corn and corn-soybean rotation. *Transactions of the ASAE*.

[37] Facelli JM, Pickett STA (1991). Plant litter: light interception and effects on an old-field plant community. *Ecology*.

[38] De Villalobos AE, Peláez DV (2001). Influences of temperature and water stress on germination and establishment of *Prosopis caldenia* Burk. *Journal of Arid Environments*.

[39] Gross KL (1984). Effects of seed size and growth form on seedling establishment of six monocarpic perennial plants. *Journal of Ecology*.

[40] Wilsey BJ, Polley HW (2003). Effects of seed additions and grazing history on diversity and productivity of subhumid grasslands. *Ecology*.

[41] Eckstein RL, Donath TW (2005). Interactions between litter and water availability affect seedling emergence in four familial pairs of floodplain species. *Journal of Ecology*.

[42] Eckstein RL, Ruch D, Otte A, Donath TW (2012). Invasibility of a nutrient-poor pasture through resident and non-resident herbs is controlled by litter, gap size and propagule pressure. *PLoS ONE*.

[43] Pérez-Ramos IM, Gómez-Aparicio L, Villar R, García LV, Marañón T (2010). Seedling growth and morphology of three oak species along field resource gradients and seed mass variation: a seedling age-dependent response. *Journal of Vegetation Science*.

[44] Nagashima H, Hikosaka K (2012). Not only light quality but also mechanical stimuli are involved in height convergence in crowded *Chenopodium album* stands. *New Phytologist*.

[45] Moles AT, Ackerly DD, Tweddle JC (2007). Global patterns in seed size. *Global Ecology and Biogeography*.

[46] Donath TW, Eckstein RL (2010). Effects of bryophytes and grass litter on seedling emergence vary by vertical seed position and seed size. *Plant Ecology*.

[47] Burmeier S, Donath TW, Otte A, Eckstein RL (2010). Rapid burial has differential effects on germination and emergence of small- and large-seeded herbaceous plant species. *Seed Science Research*.

[48] Westoby M, Leishman M, Lord J (1996). Comparative ecology of seed size and dispersal. *Philosophical Transactions of the Royal Society B: Biological Sciences*.

[49] Westoby M, Falster DS, Moles AT, Vesk PA, Wright IJ (2002). Plant ecological strategies: some leading dimensions of variation between species. *Annual Review of Ecology and Systematics*.

[50] Zanne AE, Chapman CA, Kitajima K (2005). Evolutionary and ecological correlates of early seedling morphology in east African trees and shrubs. *The American Journal of Botany*.

